# Muscular and brain cysticercosis

**DOI:** 10.1590/0037-8682-0439-2023

**Published:** 2023-10-20

**Authors:** Luã Portela, Tânia Marchiori Cardoso, Fabiano Reis

**Affiliations:** 1 Universidade Estadual de Campinas, Faculdade de Ciências Médicas, Departamento de Anestesiologia, Oncologia e Radiologia, Campinas, SP, Brasil. Universidade Estadual de Campinas Faculdade de Ciências Médicas Departamento de Anestesiologia, Oncologia e Radiologia Campinas SP Brasil; 2 Universidade Estadual de Campinas, Faculdade de Ciências Médicas, Departamento de Neurologia, Campinas, SP, Brasil. Universidade Estadual de Campinas Faculdade de Ciências Médicas Departamento de Neurologia Campinas SP Brasil

**FIGURE 1:**
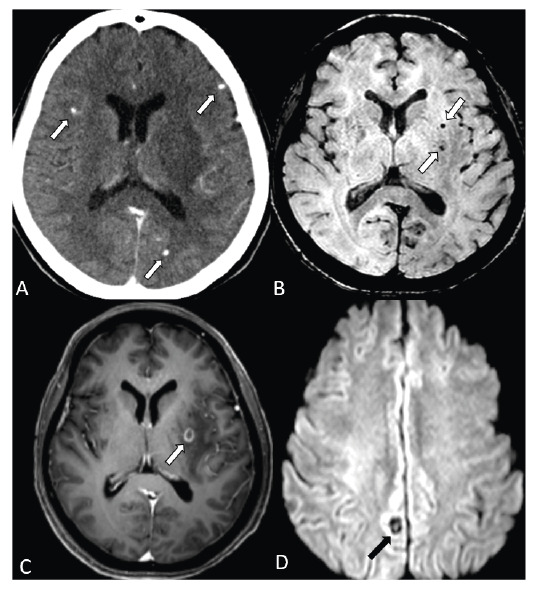
**(A)** Axial computed tomography reveals multiple sub-centimeter calcified lesions (arrows) predominantly throughout the supratentorial brain. **(B)** Axial susceptibility-weighted imaging (SWI) demonstrates multiple round hypointense foci, consistent with calcifications (arrows). **(C)** Axial contrast-enhanced T1-weighted image (WI) reveals a cystic ring-enhancing lesion (arrow) with perilesional edema in the left putamen. **(D)** Axial diffusion-weighed imaging (b=1000) reveals a hyperintense eccentric dot (scolex, arrow) indicative of neurocysticercosis in the colloidal vesicular stage.

A previously healthy 45-year-old woman presented with seizures. Computed tomography and subsequent magnetic resonance imaging (MRI) revealed calcified brain lesions (Figure 1).

MRI revealed multiple cerebral ring-enhancing lesions with cystic components, a scolex, and vasogenic edema (Figure 1). 

**FIGURE 2:**
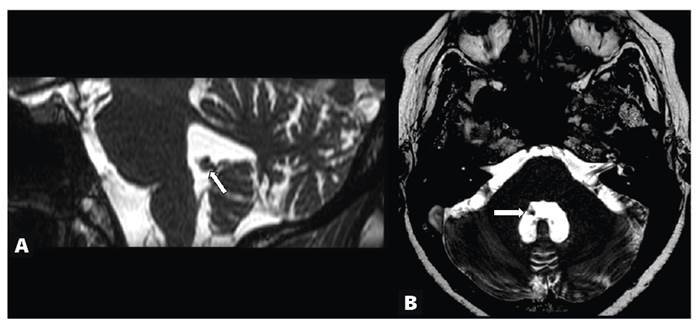
Sagittal and axial fast imaging, employing steady-state acquisition images **(A and B)** demonstrate an isointense lesion in the lower portion of the roof of the fourth ventricle (arrow). Steady-state free precession sequences help identify intraventricular cysts.

Fast imaging, employing a steady-state acquisition MRI sequence also revealed lesions in the fourth ventricle, indicating the racemose form of extra-parenchymal neurocysticercosis (NCC) (Figure 2). The racemose form, which requires prolonged treatment, is associated with increased morbidity and mortality and may lead to cerebrovascular complications^1^^,^^2^. NCC was considered the cause for the epilepsy, being among the most common causes of structural epilepsy^3^.

**FIGURE 3:**
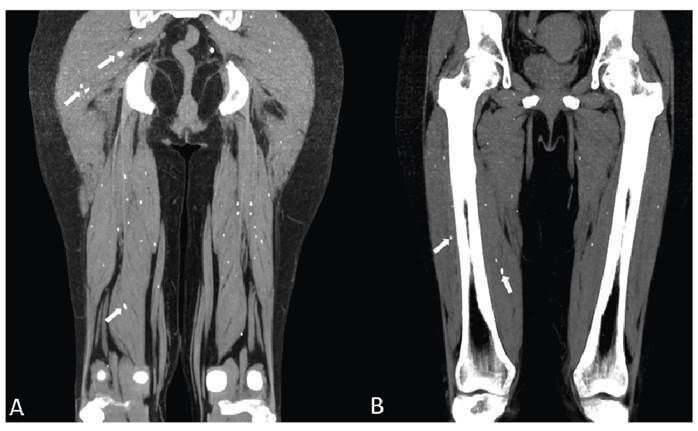
Coronal **(A and B)** non-contrast-enhanced computed tomography with maximum intensity projection reveals multiple elongated and oval-shaped calcified lesions within the hip and thigh muscles.

Rice-like ovoid calcifications resembling a starry sky were identified in the muscles (Figure 3), suggestive of cysticercosis^4^. The muscular form of cysticercosis is generally asymptomatic. However, three distinct types of manifestations may be observed: the myalgic, myopathic type; the nodular or mass-like type; and the rare pseudohypertrophy type^5^. 

If rice- or starry sky-like muscular calcifications are detected, cysticercosis must be suspected^6^. Even in patients without systemic or neurological manifestations, this finding is indicative of this diagnosis. 

This case highlights the association of NCC with the muscular form of cysticercosis and its peculiar imaging findings in a patient with epilepsy beginning in adult life.
